# Supplementation of papaya leaf juice has beneficial effects on glucose homeostasis in high fat/high sugar-induced obese and prediabetic adult mice

**DOI:** 10.1186/s12906-023-04320-1

**Published:** 2024-01-03

**Authors:** Benard B. Nyakundi, Marisa M. Wall, Jinzeng Yang

**Affiliations:** 1 Department of Human Nutrition, Food and Animal Sciences, University of Hawaii at Manoa, Honolulu, Hawaii 96822 USA; 2 Daniel K. Inouye U.S. Pacific Basin Agricultural Research Center, USDA-ARS, Hilo, HI 96720 USA

**Keywords:** Prediabetes, Papaya leaf, Blood glucose, Diabetes, Insulin sensitivity

## Abstract

Prediabetes is characterized by a cluster of glycemic parameters higher than normal but below the threshold of type 2 diabetes mellitus (T2DM). In recent years, phytochemical-rich plant extracts have gained popularity as therapeutic agents for metabolic disorders. This study investigated the effects of papaya leaf (PL) juice supplementation on blood glucose levels in diet-induced obese and prediabetic adult mice. B65JL F1 mice (*n* = 20) at 12–14 months old were fed a high fat/sugar diet (HFHS) for 120 days. Mice were switched to restricted rodent chow of 3 g feed/30 g body weight/day, supplemented with 3 g/100 mL PL juice for 30 days. HFHS diet remarkably increased fasting plasma glucose levels from 114 ± 6.54 mg/dL to 192.7 ± 10.1 mg/dL and body weight from 32.5 ± 1.6 to 50.3 ± 4.1 g. HFHS diet results in hyperglycemia, insulin resistance, hyperlipidemia, and liver steatosis. The combination of PL juice and restricted diet significantly reduced body weight and fasting blood glucose levels to 43.75 ± 1.4 g and 126.25 ± 3.2 mg/dl, respectively. Moreover, PL juice with a restricted diet significantly improved lipid profile: cholesterol from 204 to 150 mg/dL, LDL-c from 110.4 to 50 mg/dL, and triglyceride from 93.7 to 60 mg/dL. Additionally, PL juice combined with a restricted diet significantly reduced adiposity, reversed fatty liver, and restored skeletal muscle Glut4 and phosphorylated (p-AKT (ser473). This study demonstrated that supplementation of PL juice with a restricted diet was more effective than a restricted diet alone in reversing major symptoms related to prediabetic and obesity conditions.

**Supplementary Information:**

The online version contains supplementary material available at 10.1186/s12906-023-04320-1.

## Introduction

Type 2 diabetes mellitus (T2DM) is characterized by hyperglycemia and disturbances of carbohydrate, fat, and protein metabolisms associated with insulin resistance or/and insulin deficiency. Obesity, sedentary lifestyles, and genetic disposition are among the risk factors associated with T2DM [1]. Uncontrolled diabetes can lead to systemic failures such as nephropathy, neuropathy, retinopathy, and cardiovascular complications. Besides, it is associated with increased infection risks due to slow wound healing and some cancer types [2]. According to the National Diabetes Statistics Report 2020, 10.5% of Americans were diabetic. Surprisingly, 23% of adults whose blood laboratory testing results would meet diabetes criteria did not report or were unaware of their conditions, and diabetes prevalence was found to be higher among those aged 45 and above [3]. Diabetes costs Americans $237 billion in direct medical expenses and $90 billion in lost productivity annually. Moreover, 61% of Medicare healthcare costs for seniors over 65 are associated with diabetic complications [4]. Although T2DM cannot be cured currently, changes in lifestyle habits can delay or prevent it, together with diet and medication [5]. Pharmacological hypoglycemic drugs have side effects such as nausea, diarrhea, and hypoglycemia, are also expensive for middle and low-income patients. Additionally, it is difficult to take regularly with adverse effects in clinical applications [6]. Developing affordable alternative hypoglycemic agents with minimal side effects can reduce diabetes-related complications and improve the quality of life in diabetic patients [7].

The use of plant-derived antidiabetic agents such as nutraceuticals, bioactive compounds, and supplements has gained popularity in recent years [8]. The mechanism through which plant-based compounds exert antihyperglycemic effects involves enhancing glucose uptake, regeneration of pancreatic alpha cells, restoration of glucose homeostasis, and inhibition of α-amylase and α-glucosidase enzymes [9–11]. Besides, they do not cause as many side effects as oral hypoglycemic drugs. *Carica papaya*, commonly known as papaya is considered therapeutic in traditional medicine. Various parts such as leaves, seeds, ripe and unripe fruits, roots, and juice were used to treat illnesses. Papaya's medicinal benefits include antioxidant, anti-obesity, antibacterial, antifungal, and hypotensive properties [12]. Recent studies in animals have shown that papaya leaf (PL) extracts regulate blood sugar levels. For instance, 5 mg/kg of PL juice extract combined with different concentrations of oral hypoglycemic agents produced significant (*P* < 0.05) anti-hyperglycemic effects on Alloxan-induced diabetic rats [13]. Moreover, PL ethanolic extract exhibits hypoglycemic effects, regeneration of pancreatic beta cells, and recovery of kidney cuboids in streptozotocin (STZ)-induced diabetic mice [14]. Furthermore, in STZ-induced diabetic rodents, PL extracts improved diabetic symptoms by increasing nitric oxide, preserving hepatocyte integrity, and preventing glycogen and lipid accumulation in the liver [15]. PL extract phytochemicals are implicated in reduction of hyperglycemia by restoring the physiological antioxidant system. Steroids, quinones, flavonoids, saponins, proanthocyanins, tocopherol, and benzyl isothiocyanate are among the phytochemicals contained in papaya extract [16]. Animal experiments have shown, however, that whole-plant extracts have antihyperglycemic effects rather than single compounds. Furthermore, these effect has been demonstrated in drug-induced diabetic models, a representative of type 1 diabetes mellitus, which accounts for about 2% of all diabetes cases [17]. Almost 95% of diabetes are classified as Type 2 Diabetes, which is associated with obesity, diet and aging [3]. The purpose of this study was to investigate the antihyperglycemic properties of PL juice supplementation in high-fat/high sugar-induced obese and prediabetic adult mice. Considering the prevalence of T2DM among adults over 50 years of age, this mouse model at more than 12–14 months old is suitable for testing whether PL juice can reverse diet-induced prediabetes among adults.

## Materials and methods

### Animal

Animals were housed in the Small Animal Facility (SAF) at the University of Hawaii Manoa and maintained at 22 °C under a 12-h light/12-h dark cycle. Before the study period, animals were raised on a free access rodent diet (crude protein 18%, crude fat 6% fiber 7%) of Mazuri 5M30 for Rodent Breeder. The experiments were conducted on wild-type (B65JL F1) male and female mice (12–14 months old) weighing between 33 and 38 g. All experimental protocols related to animal work were reviewed and approved by the University of Hawaii IACUC under protocol number 20–3427-3. Animal care and uses for the study is in accordance with ARRIVE guidelines (https://arriveguidelines.org).We induced obesity and prediabetes by using a high-fat diet (D12451, 45% kcal fat, metabolizable energy 4.73 kcal/g, Research Diet Inc), and drinking water enriched with 42 g/L of cane sugar and fructose in a ratio of 1:1 [18]. The diet and water were supplied ad libitum for 120 days. Animals with an average body weight of ≥ 44 g with a fasting plasma glucose of ≥ 135 mg/dL were used for the study. Mice aged 12–14 months are equivalent to 55–60-year-old humans [19]. The obese and hyperglycemic mice were divided into two groups of 10 each. The first group was given a restricted normal diet of 3 g/day/30 body weight [20]. The second group was fed a restricted diet (3 g/day/30 body weight) supplemented with 3 g/100 mL of PL juice for 30 days. Six (6) untreated mice were used as a control group. The weight of the mice was measured on days 0,7,14,21,30 of PL juice supplementation, while fasting plasma glucose (FPG) was measured on day 0, 15, and 30 from the restricted diet feeding. An assessment of plasma lipids and an examination of liver and muscle tissues were conducted. No chemical or drugs were used in animals, including drugs for anesthesia purposes.

### Carica papaya leaf processing

C. papaya leaves were harvested from a local farm with permission from the plant owner, identified using the Plant Identifier (Pl@ntNet) application, and authenticated by papaya fruit and seed by Dr. Jinzeng Yang at the University of Hawaii at Manoa (serial number 2022/CP/001)*.* Leaves were sprayed with 75% ethanol, and washed in distilled water, and their moisture content was determined. 250 g of leaves were blended in 500 mL of tap water by a blender. The homogeneity was sieved through a cloth strainer and stored in a -20 °C freezer. 3 g/100 mL PL was administered to experimental mice [15]. We used water as a green solvent for papaya juice extraction. The toxicity of green solvents is generally low. To avoid toxicity of drinking PL juice by the mice, one week before the experiment, we fed a few mice PL juice at a concentration five times greater than that used in the experiment, and the mice appeared normal. Therefore, we used 3 g /100 ml PL for the feeding trial.

### Fasting blood glucose and insulin sensitivity test

To determine fasting plasma glucose (FPG) the mice were fasted for 8 h overnight with access to drinking water. Insulin sensitivity was performed by intraperitoneally injecting 0.75U/kg body weight of human insulin Novolog flexPen (Plainsboro, NJ). At 0, 30, 45, 60, and 120 min post-insulin injection blood was collected from the tail nick, and plasma glucose levels were determined by a glucometer (OneTouch UltraMini).

### Lipid profile and adiposity

To determine the lipid profile, we collected blood drops from the tail vein. The CardioChek Analyzer (Whitestown, IN) was used to measure total cholesterol, high-density lipoprotein, triglycerides, and calculated low-density lipoproteins. Epididymal and retroperitoneal adipose tissues were dissected and weighed after mice were euthanized with CO_2_ asphyxiation.

### Liver and muscle histological examination

Liver and muscle tissues were collected from four mice of each group; normal, obese, PL & diet and diet alone and fixed in 10% neutral buffer formalin for 24 h. Samples were transferred to 70% ethanol, paraffin-embedded, and sectioned at the Histopathological Core Facility of UHM. The liver sections were stained with Periodic Acid Schiff (PAS), whereas the muscle sections were stained with hematoxylin and eosin (H&E). The sections were examined with an Olympus BX43 microscope.

### Western blot

For detecting phosphor-Akt Ser473, Gastrocnemius samples were collected 8 min post insulin intraperitoneal injection 10U/kg body weight as described by [21]. Muscle tissues for GLUT 4 (*n* = 5) was collected as well and homogenized in freshly prepared lysis buffer (150 sodium chloride, 1.0% NP-40, 50 Tris pH 8.0, protease inhibitor mini tablets cocktail, 1 mM PMSF and 10 mM sodium fluoride) and centrifuged at 15000 g for 15 min at 4 °C. Protein concentration was determined by the BCA Protein Assay kit (Pierce, IL, USA). 60 μg of protein were separated by 12% SDS-PAGE then transferred to nitrocellulose membranes and blocked in 5% non-fat milk. Primary antibodies; GLUT4(IF8) (#2213, 1:1000), p-Akt (Ser473) (#4058, 1:1000) Akt(#9272, 1:1000), and β-Actin (#3700, 1:1000) were purchased from Cell Signaling Technology (CST) (Danvers, MA, USA) Secondary antibody, Ant-mouse HRP-linked (#7076P2, 1:3000) CST and Ant-rabbit IgG HRP-conjugated (#HAF008, 1:3000) R&D Systems (Minneapolis, MN, USA). Protein signals were developed by an enhanced chemiluminescence kit (Thermos Scientific Rockford, Illinois, USA). Image blots were acquired by digital image iBright 1500 Invitrogen (Thermo Fisher Scientific Waltham, MA USA) and quantified with ImageJ, NIH, Bethesda, Maryland.

### Statistics analysis

Statistical analysis was performed using GraphPad Prism 5 statistical package (GraphPad Software, San Diego MA, USA). Using skewness, kurtosis, and Leven's test, we determined normality and homogeneity of variances**.** All data on the effects of PL juice supplementation on weight, adipose tissues, and each set of the mean from insulin sensitivity test were analyzed and presented in means ± SEM with one- or two-way analysis of variance (ANOVA) followed by Turkey’s test, statistics analysis. Significance was determined at *P* < 0.05.

## Results

### Effect of high-fat diet/high sugar (HFHS) diet on body weight and glucose levels

**Fig. 1:**
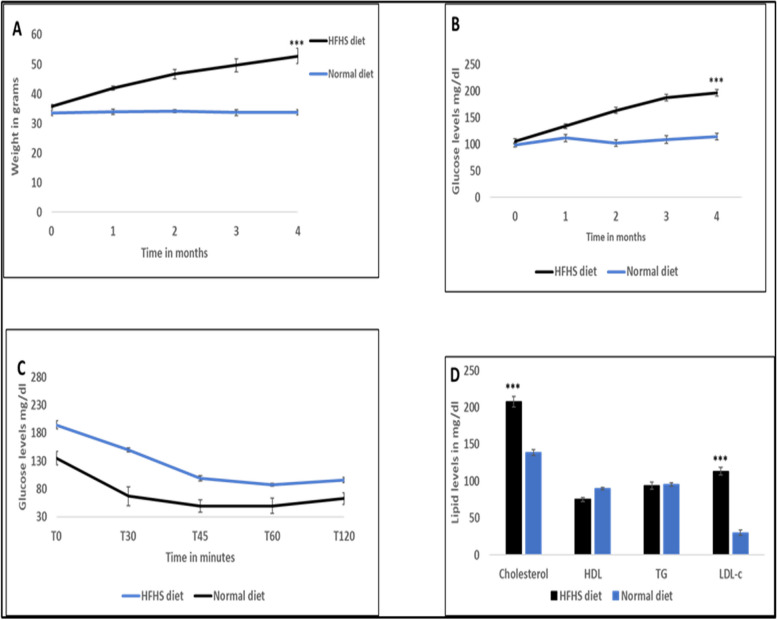
High fat/ high sugar diet (HFHS) induces hyperglycemia and obesity: **A** Weight progress through 4 months of HFHS diet. **B** Fasting plasma glucose levels during the HFHS feeding period. **C** Comparing insulin sensitivity between the normal and HFHS groups and (D) Lipid profile, cholesterol, HDL (high-density lipoprotein), TG (triglycerides), LDL-c (calculated low-density lipoprotein levels between normal and HFHS mice data shown in means SEM, *N* = 20 ***P < 0.001, **P < 0.01 *P < 0.05

Initial measurements were taken, and the mice were subjected to the HFHS diet for four months. The control group was fed with normal rodent chow diet. Body weights and fasting plasma glucose (FPG) levels were measured monthly. There was a significant increase in body weight of the HFHS group (52.8 ± 2.68) compared to the control group (33.78 ± 0.90) (*p* < 0.001) (Fig. 1A). Fasting plasma glucose levels increased remarkably in the HFHS group (196.4 ± 6) compared to the normal diet group (114 ± 6.54) (*p* < 0.001) (Fig. 1B). While glucose levels were relatively higher in the HFHS diet group after insulin injection, there was no significant difference between the two groups in insulin sensitivity (Fig. 1C). However, total cholesterol and calculated low-density lipoprotein were significantly higher in the HFHS group (207.75 ± 7.38 and 113.47 ± 5.24) respectively, compared to the normal diet group (138.57 ± 4.08 and 29.85 ± 3.06) (*P* < 0.001**)** (Fig. 1D).

### Effect of PL juice on fasting plasma glucose and insulin sensitivity

**Fig. 2:**
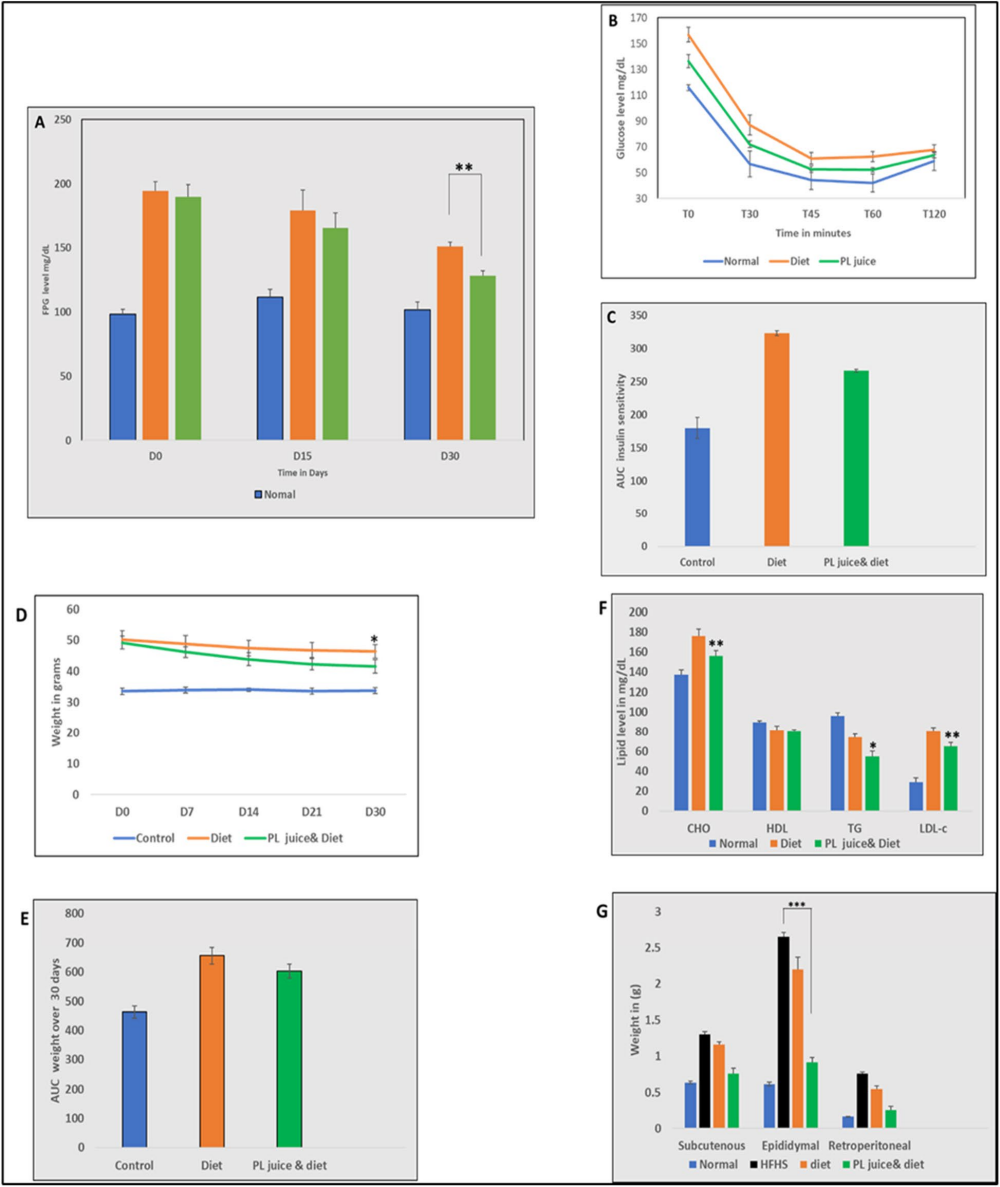
PL supplementation is antihyperglycemic and increases insulin sensitivity. **A** Fasting plasma glucose was measured Day (D0, D15 and D30) after the introduction of PL juice with restricted diet. **B** 0.75 I.U of insulin was to the experimental mice and glucose level was measured at T(minutes) 0, 30, 45, 60, and 120. **C** Area under curve (AUC) for insulin sensitivity test. PL juice supplementation reverses obesity and improves lipid profile (**D**) Weight change after 30 days of PL juice supplementation. **E** Area under curve for weight changes. **F** Plasma lipid profile after PL juice supplementation total cholesterol (CHO), high-density lipoprotein (HDL), triglycerides (TG), calculated low-density lipoproteins (LDL-c). (G) Fat pad distribution on the normal, obese, restricted diet and PL juice supplemented groups data shown in means S.E.M ***P < 0.001, **P < 0.01, *P < 0.05

After HFHS treatment, mice were randomly divided into two groups of 10 mice each, restricted normal fat diet alone and restricted diet supplemented with PL juice as previously described. During the 30 days of the interventional treatment, fasting plasma glucose was measured before treatment (D0), middle, and end of treatment (Fig. 2A). Both restricted diet and PL juice-supplemented groups showed substantial reductions in FPG. However, there was a significantly lower FPG level in the PL juice-supplemented group (128.12 ± 3.9) compared to the lone-restricted diet group (150.8 ± 3.4) (*p* < 0.01). Insulin sensitivity in both the PL juice-supplemented with restricted diet and restricted diet alone groups was comparable to the normal group (66.63 ± 2, 67.8 ± 3.7, and 59 ± 7.3 mg/dL) respectively (Fig. 2B). The area under the curve (AUC) insulin sensitivity in the restricted diet group was slightly greater than the PL juice-supplemented group (Fig. 2C).

### Effect of PL juice on body weight, lipid profile, and fat tissue distribution

To investigate the effect of PL juice on weight, we recorded the weight of the mice after the introduction of PL juice on days (D) 0,7, 14, 21, and 30 subsequently. We further recorded lipid profile readouts from tail vein obtained blood samples. Mice were sacrificed at the end of the experiment and subcutaneous, epididymal, and retroperitoneal adipose tissues were collected and weighed. PL juice supplemented with a restricted diet significantly reduced the body weight *P* < 0.05 compared to the group fed with a restricted diet alone at the end of the 30th day (41.56 ± 2.15 vs 46.4 ± 2.14 g) (Fig. 2D). Although the area under the curve for the PL juice-supplemented group was slightly lower than the lone-restricted diet group, the differences were not significant (Fig. 2E). PL juice supplementation with a restricted diet significantly reduced total cholesterol levels compared to a restricted diet alone (145.8 ± 5.46 vs 176.2 ± 6.96 mg/dL) *p* < 0.01. We did not detect any difference in the high-density lipoprotein levels (HDL). Interestingly, triglyceride (TG) levels were remarkably reduced in the PL juice-supplemented group (*p* < 0.05). Additionally, calculated low-density lipoprotein (LDL-c) levels in the PL-supplemented group were substantially reduced compared to the lone-restricted diet group (55 ± 4.1 vs 80.04 ± 4.04 mg/dL) *p* < 0.01 (Fig. 2F). Compared to HFHS mice, both PL-supplemented and lone-restricted diet groups displayed reduced adiposity. The percentage of fat pad decrease was higher in the PL juice-supplemented group compared to mice fed a restricted diet alone, including subcutaneous fat (41% vs. 10%), epididymal fat (65% vs. 17%), and retroperitoneal fat (65% vs. 27%) (Fig. 2G).

### Liver and muscle histopathology

**Fig. 3:**
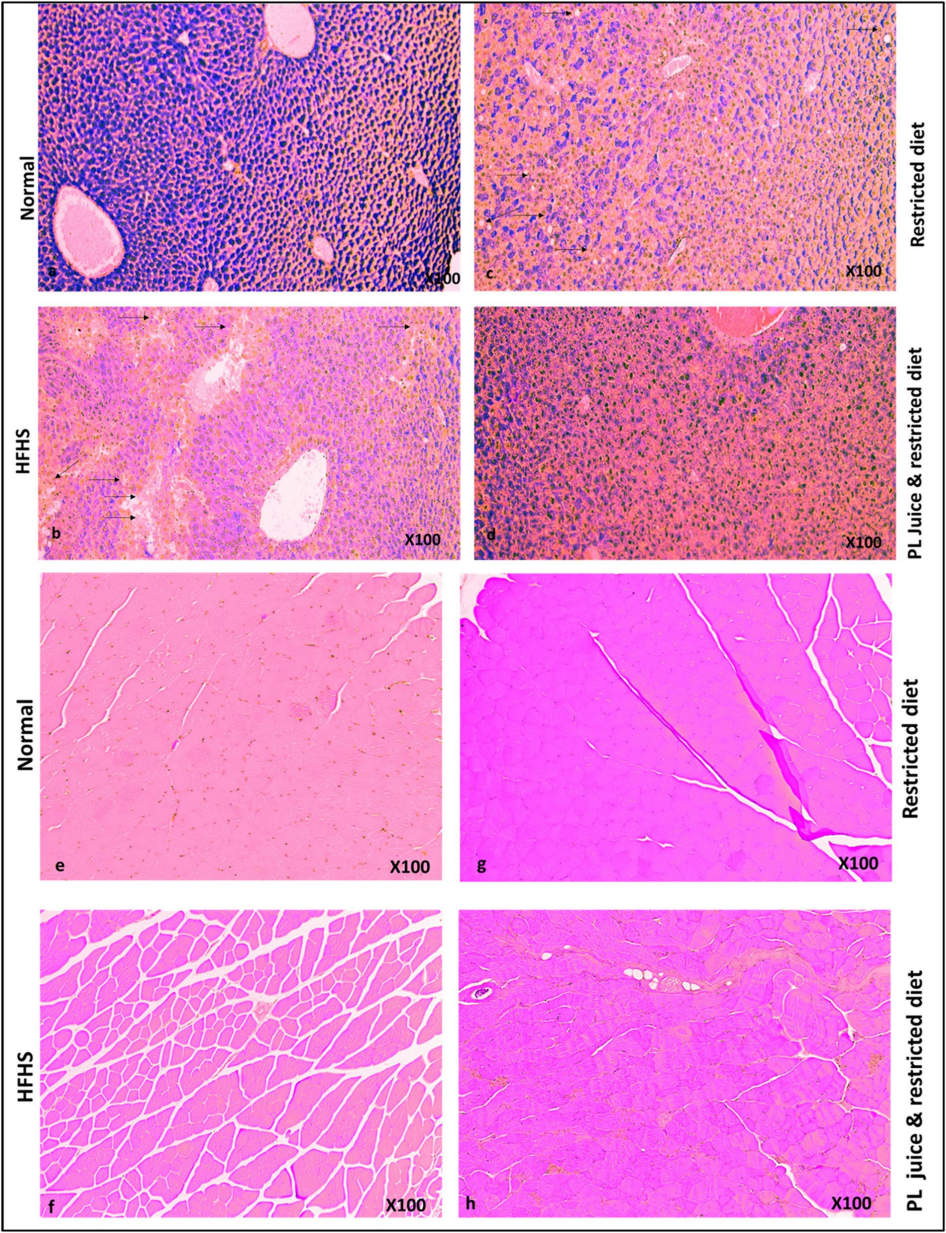
PAS (Periodic Acid Schiff) staining of liver tissue slices from normal, High fat/high sugar (HFHS) diet, and PL juice with diet (**a**,**b**,**c**,**d**). PL juice and a restricted diet reduced liver fat accumulation and glycogen deposition. Representatives of H&E-stained gastrocnemius muscle tissues from Normal, HFHS restricted diet and PL juice with restricted diet (**e**,**f**,**g**, and **h**) all muscles showed normal structure *N* = 4

To assess the effect of PL supplementation on the liver and muscle tissues of the HFHS diet, the mice were sacrificed at the end of the 30th day of PL juice supplementation. The liver and gastrocnemius tissues were sampled, fixed in 10% neutral buffer formalin, and paraffin-embedded sections were prepared. PAS-stained normal liver cells (Fig. 3a) contrasted with HFHS diet groups exhibited multiple streaks of fat accumulation (Fig. 3b). There was a remarkable reduction in fat accumulation after a restricted diet (Fig. 3c), which was more pronounced in the PL-supplemented group with reduced glycogen deposits (Fig. 3d). All muscle sections showed normal structure (Fig. 3 e,f,g and h).

### Effect of PL juice supplementation on insulin signaling

**Fig. 4:**
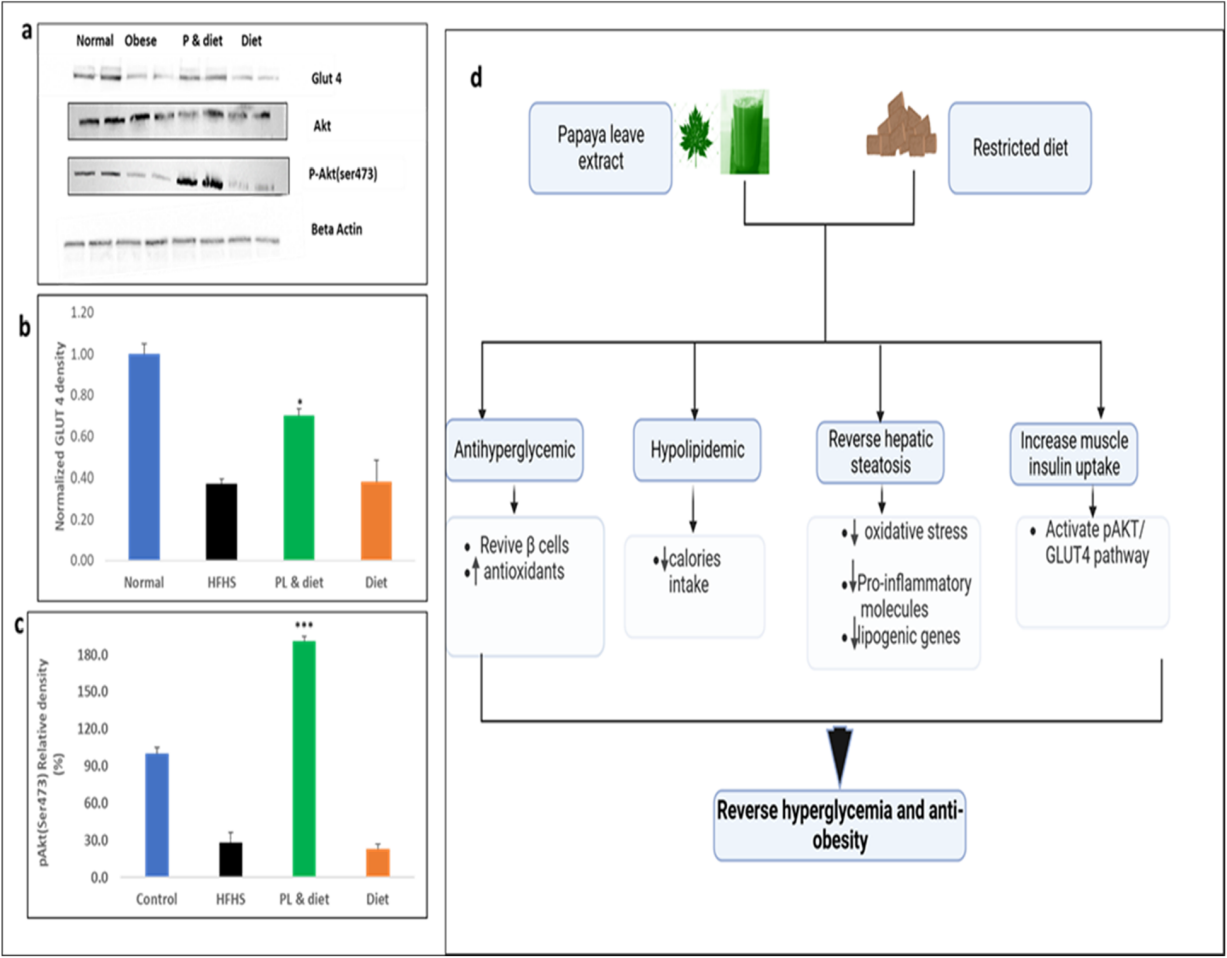
Effect of PL juice supplementation on skeletal Muscle expression of GLUT 4 phosphorylated AKT (p-Akt(ser473muscles. Gastrocnemius muscle samples were collected from experimental mice and for pAKT(ser473) mice were injected with insulin and 8 min post-injection samples were collected for western blot. The blots are representative of 3 independent experiments (**a**). The relative density of GLUT4 to total β-Actin (**b**) Quantitation of relative band density percentage of p-Akt (Ser473) to Akt (**c**) values are represented in SEM) *P < 0.05, ***P < 0.01. Graphical abstract of the mechanism of combined PL juice supplementation with restricted diet (**d**)

In response to insulin stimulation and AKT, GLUT4 is translocated to the plasma membrane and enhances insulin-stimulated glucose uptake in muscle tissues. To investigate the mechanism through which PL juice lowers blood sugar levels, we examined muscle protein GLUT 4, AKT, and p-AKT (ser473). PL juice-supplemented group exhibited relatively significantly higher levels of Glut4 expression (*p* < 0.5) in the gastrocnemius muscle tissues than the group that was fed with a restricted diet alone. Phosphorylated Akt levels were more pronounced at 180% relative expression to Akt (*p* < 0.01) (Fig. 4 a, b and c).

## Discussion

Prediabetes occurs before the manifestation of diabetic symptoms. Elevated fasting blood glucose, impaired glucose tolerance, and increased glycated hemoglobin (A1C) readouts are used to determine the onset of prediabetic conditions. We investigated the effects of supplementation of PL juice on reversing prediabetic symptoms in diet-induced obesity and hyperglycemia adult mice. Mice were fed a 45% kcal fat diet with 42 g/L of fructose and sucrose for 16 weeks. Adult mice fed high-fat/high-sugar diets developed obesity, hyperglycemia, and other prediabetic-related symptoms such as the onset of insulin resistance, increased total cholesterol, and low-density lipoprotein. Consistent with previous studies showing that diets rich in saturated fats and carbohydrates contribute to overweight, glucose intolerance, and type 2 diabetes [22, 23]. Furthermore, high fructose consumption can increase insulin resistance, low-density lipoprotein, triglyceride levels, and body fat levels leading to obesity [24]. A model of type 1 diabetes, Alloxan/Streptozotocin-induced diabetic mice, has been used to study PL's effect on diabetes. To mimic the increased susceptibility of the aged demographic towards diet-induced prediabetes we used mice between 12 and 14 months of age, HFHS diet had similar effects on males and females (data are not shown).

Our results showed that supplementation of PL juice with the restricted diet for 30 days significantly reduced fasting plasma glucose (*p* < 0.01), compared to the group that fed on a restricted diet alone. Diet restriction reduces calories without depriving the normal function of body metabolism. This approach has been effective in managing diabetes, delaying age-related metabolic disorders, and improving metabolic health [25, 26]. However, diabetes can be controlled more effectively through comprehensive approaches such as a diet and exercise regimen or by combining a diet with oral herbal supplements with hypoglycemic effects [27]. We combined PL juice with a restricted diet. In our study, PL juice supplementation (3 g/100 mL in drinking water) for 30 days increased insulin sensitivity, resulting in a slightly reduced area under the curve (AUC) compared with a restricted diet alone. According to studies conducted on the hypoglycemic effects of *Artemisia dracunculus* and Berberine herb, a substantial reduction in AUC insulin sensitivity was observed [28, 29]. While HFHS did not cause a significant difference in insulin resistance, it increased the baseline level of resistance, which was restored by PL juice supplementation. Compared to the restricted diet alone, PL juice supplementation reduced body weight with a slightly lower AUC in the PL juice supplementation group (Fig. 2D and E). PL juice supplementation significantly reduced total cholesterol, LDL, and plasma TG levels (*P* < 0.05) and (*P* < 0.01) (Fig. 2F). Furthermore, epididymal and retroperitoneal fat pads in the PL juice-supplemented group were significantly reduced (*p* < 0.001) and (*p* < 0.05) respectively (Fig. 2G). The anti-obesity and hypolipidemic properties of PL juice supplementation in our study are consistent with those observed in other studies on animal models [30]. Papaya contains many phytochemicals that act as antioxidants and anti-inflammatory agents, including phenolic compounds, benzyl isothiocyanate, and tocopherol [31]. PL's adiposity-reducing qualities may be attributed to its ability to reduce pro-inflammatory cytokines (interleukin-6) as well as its ability to increase serum levels of superoxide dismutase [32]. Consistently, histological examination of liver tissues revealed a reduction in glycogen storage and clearance of fat deposits in the PL juice-supplemented group (Fig. 3d). Most of the lipid accumulation in non-alcoholic fatty liver disease (NFLD) is associated with the circulating pool of free fatty acids (FFAs) in obese individuals. PL may suppress lipogenic genes such as sterol regulatory element-binding protein 1c and fatty acid synthase in diet-induced hepatic steatosis [33].

Histological observation of muscle tissues of all groups was normal (Fig. 3e, f,g,h). It is well established that the most important site of insulin-stimulated glucose utilization occurs in skeletal muscles. In response to insulin receptor activation (IRS-1), the Akt protein kinase is activated, resulting in the translocation of GLUT4 from the intracellular storage vesicles to the plasma membrane upon insulin stimulation [34, 35]. In the current study, HFHS remarkably blunted the expression of GLUT4 and phosphorylated Akt (p-Akt ser473), which is probably due to the influx of free fatty acids into the skeletal muscles, resulting in long-chain acyl-CoAs (LCACoA) formation and production of ceramide and diacylglycerol (DAG), which inhibits the insulin signaling pathway through the PI3K/AKT pathway [36]. PL juice supplementation with a restricted diet remarkably revived the insulin signaling pathway compared to the lone-restricted diet mice (Fig. 4 a). IR and GLUT4 muscle expression was elevated by ethanolic PL extracts in diabetic mice, as demonstrated by Roy et al. [37]. This effect may be due to papaya's phenols and flavonoids [30]. Phytochemicals such as gallotannins, 3-taraxerol, Astragalus polysaccharide, Berberine, and vanillic acid contribute to insulin signaling by increasing GLUT4 translocation, adiponectin secretion, PKB-Ser473 phosphorylation, decreased activity of glucokinase (GK), increased activity of glucose-6-phosphatase (G6Pase), and beta-cell stimulation [38]. Through increased antioxidants, anti-inflammatory properties, and suppression of lipogenic genes, papaya juice may augment the therapeutic effects of a restricted diet as shown in (Fig. 4d). Further research is needed to clarify whether PL's anti-hyperglycemic effect is a result of a specific bioactive component or the entire extract. Moreover, it is necessary to determine which extraction method could yield the highest bioactive compounds with the highest efficacy in regulating glucose homeostasis.

In summary, this is the first study that demonstrates PL juice supplementation to a restricted diet accelerates the reverse of high blood sugar levels and lowers cholesterol levels, triacylglycerol, and low-density lipoprotein levels in high energy-induced mice. PL supplementation significantly reduced liver fat deposition and excess epididymal adiposity when compared to a restricted diet alone. Activation of skeletal muscle insulin signaling was detected in PL juice supplementation. This study suggests that PL juice supplementation may have synergistic or complementary effects on controlling blood glucose homeostasis in prediabetes.

### Supplementary Information

**Additional file 1.**

## Data Availability

The datasets used and/or analyzed during the current study are available from the corresponding author on reasonable request.
