# Supplementary material for: Supplementation of papaya leaf juice has beneficial effects on glucose homeostasis in high fat/high sugar-induced obese and prediabetic adult mice
Source: BMC Complement Med Ther. 2024 Jan 3;24:18. doi: 10.1186/s12906-023-04320-1 (PMC10765817; doi:10.1186/s12906-023-04320-1)
Supplement: Supplementary file 1 — Additional file 1. [file 12906_2023_4320_MOESM1_ESM.pdf]

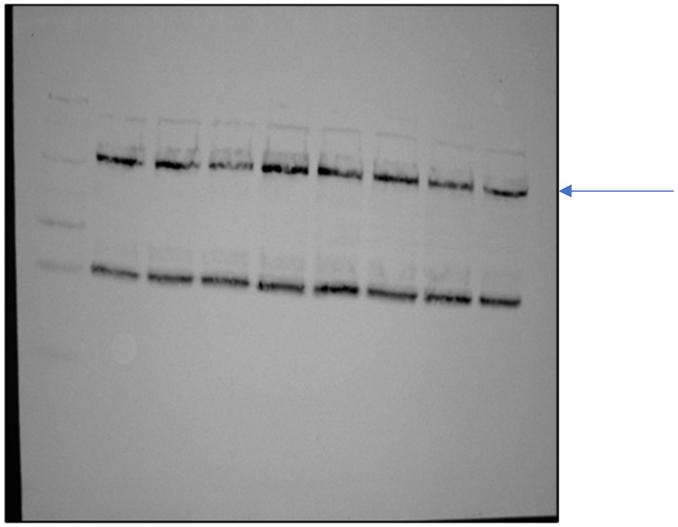

Date: 7/11/2023

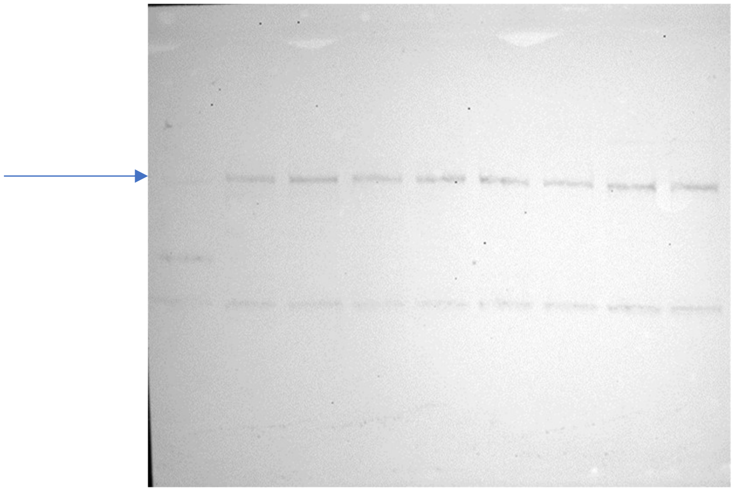

Date: 7/16/2023

- WB: primary Ab: beta Actin mouse mAb Dil 1:1000 (CST)
- Secondary Ab (anti-mouse IgG) dilution: 1:2000
- Exposition time : 3-10 seconds (Reprobe)
- Loaded sample: 60µg
- M.W 45 kDa
- Muscle: Gastrocnemius

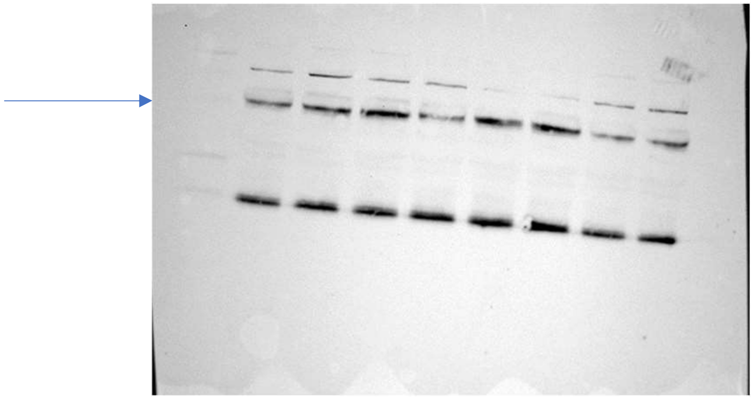

7/21/2023

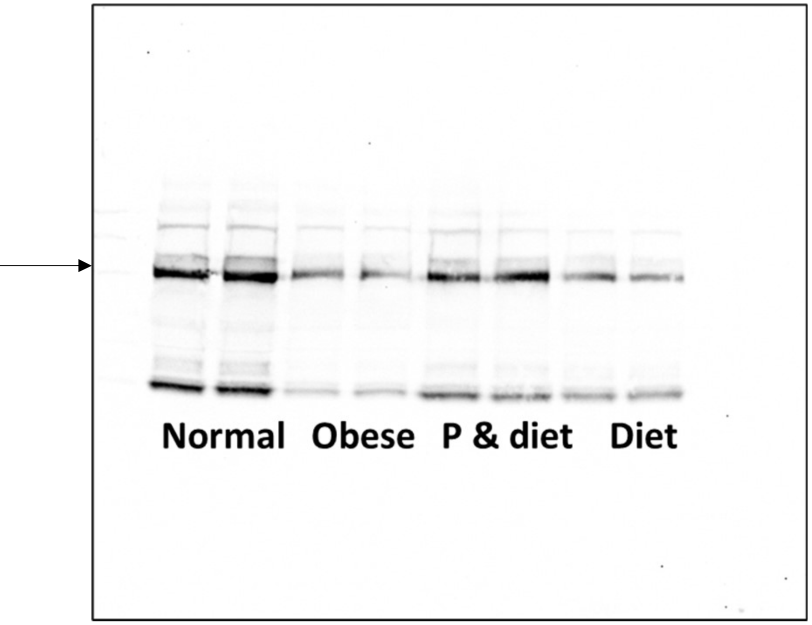

7/11/2023

- WB: primary Ab: **Glut4 (IF8)** mouse mAb Dil 1:1000 (CST)
- Secondary Ab (anti-mouse IgG) dilution: 1:2000
- Exposition time : 3-10 seconds
- Loaded sample: 60ug
- M.W 50 kDa
- Muscle: Gastrocnemius

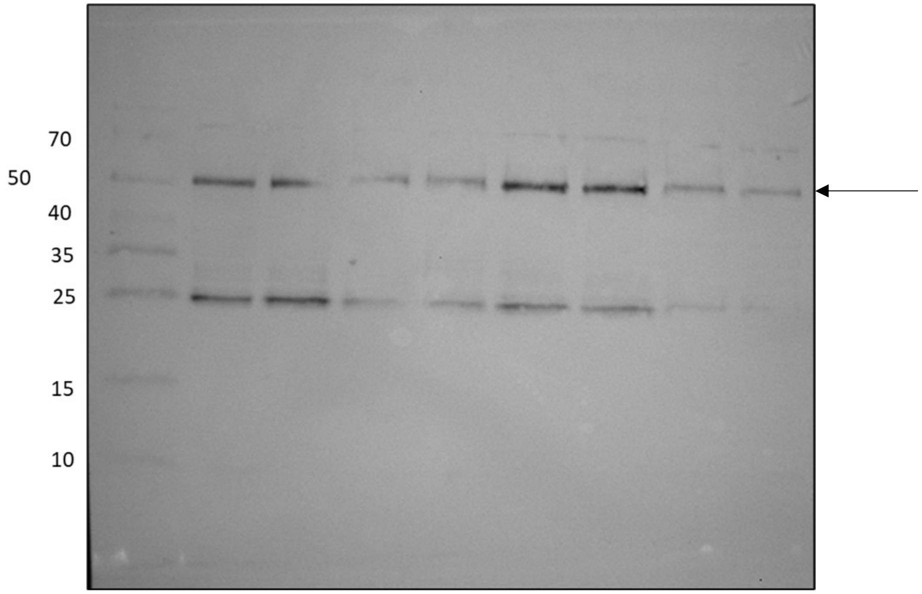

Date: 6/27/2023

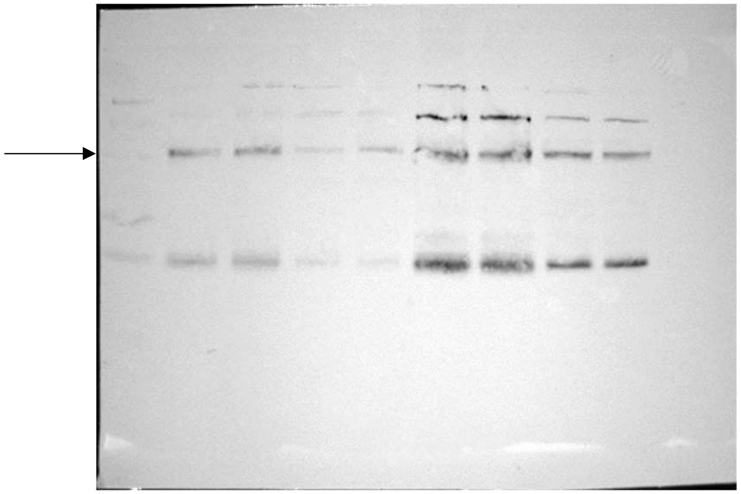

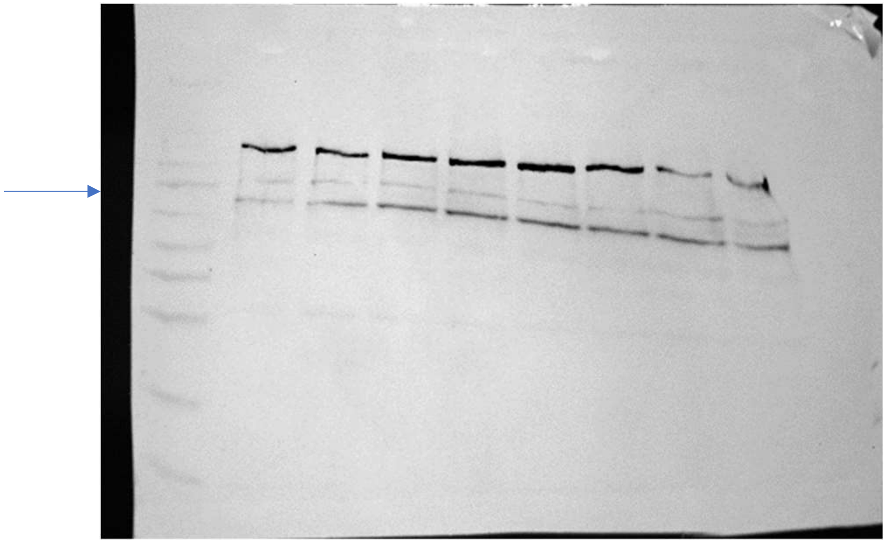

7/28/2023

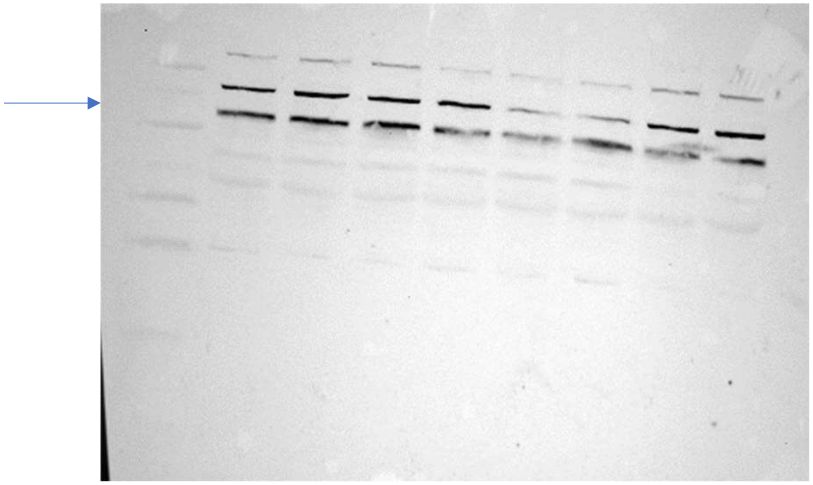

7/21/2023

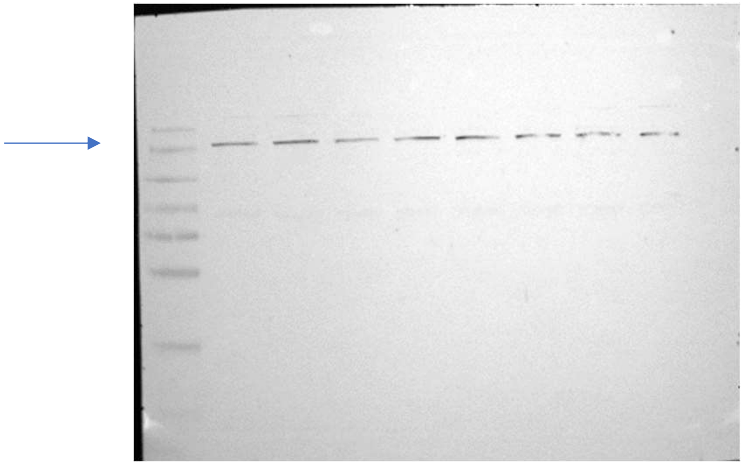

9/18/2023

- WB: primary Ab: Akt Rabbit Ab Dil 1:1000 (CST)
- Secondary Ab (anti-Rabbit IgG) dilution: 1:2000
- Exposition time :3-20 seconds
- Loaded sample: 60µg
- M.W 60 kDa
- Tissue: Gastrocnemius

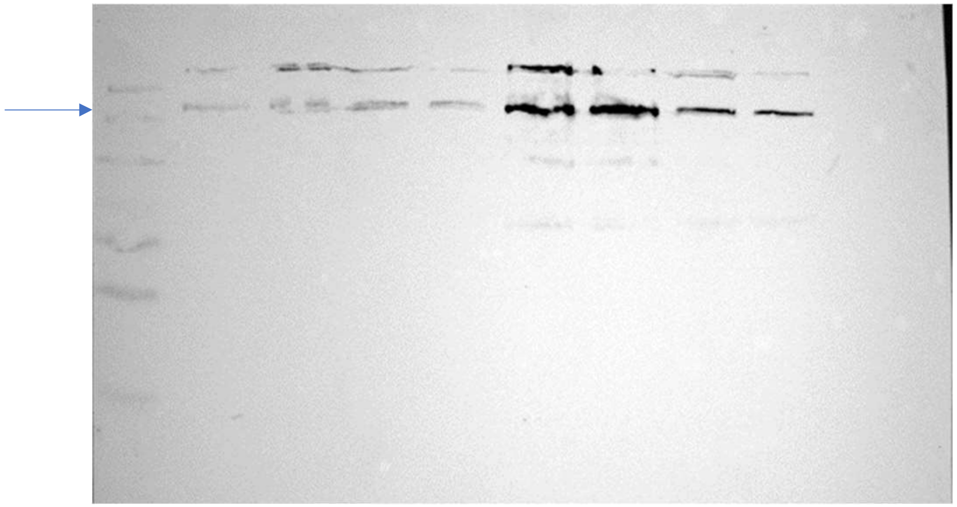

7/21/2023

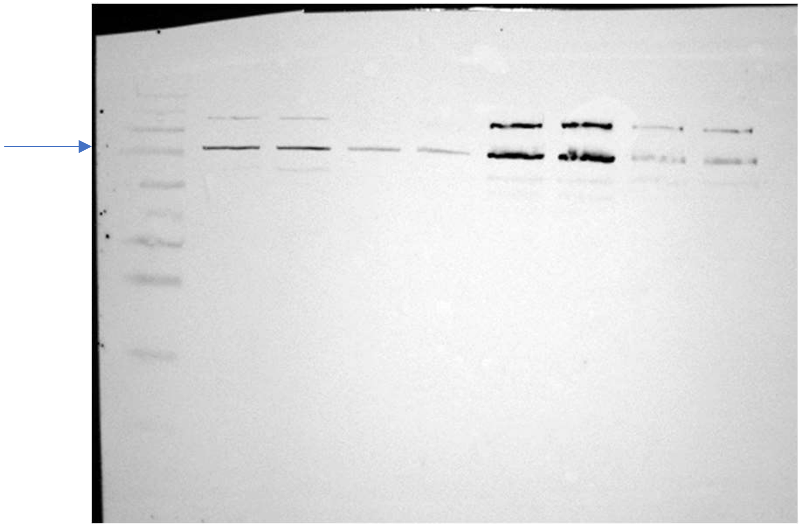

7/19/2023

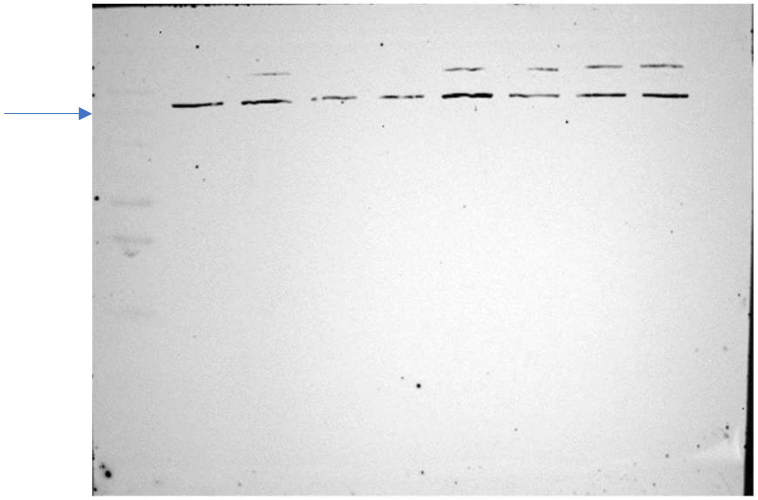

9/18/2023

- WB: primary Ab: Phosph-Akt (Ser473)(193H12) Rabbit mAb Dil 1:1000 (CST)
- Secondary Ab (anti-Rabbit IgG) dilution: 1:2000
- Exposition time :15-20 seconds
- Loaded sample: 60ug
- M.W 60 kDa
- Muscle: Gastrocnemius (Insulin injected group)
